# Initiation of condensation of toluene and octane vapours on a Si surface

**DOI:** 10.1039/d0ra01219j

**Published:** 2020-04-24

**Authors:** Sima Yaghoubian

**Affiliations:** Thermodynamics and Kinetics Laboratory, Department of Mechanical and Industrial Engineering, University of Toronto 5 King's College Road Toronto M5S 3G8 Canada sima.yaghoubian.ghouchani@utoronto.ca

## Abstract

The adsorption of toluene and octane vapours on a homogenous silicon surface was measured under steady, thermal disequilibrium conditions where a vapour at a temperature *T*^V^ is exposed to a solid surface at a lower temperature, *T*^S^. Zeta adsorption isotherm theory was used along with Gibbsian thermodynamics to examine the adsorption results analytically and to investigate the wetting conditions for these vapours. Further, from the prediction of the cluster distribution in the adsorbate, the conditions for the initiation of a liquid phase are predicted. Finally, the mechanism that determines the condensation mode of hydrocarbons on a silicon surface is investigated.

## Abstract 2

The use of the zeta adsorption isotherm in determining the condensation mode of toluene on silicon.

## Graphical abstract

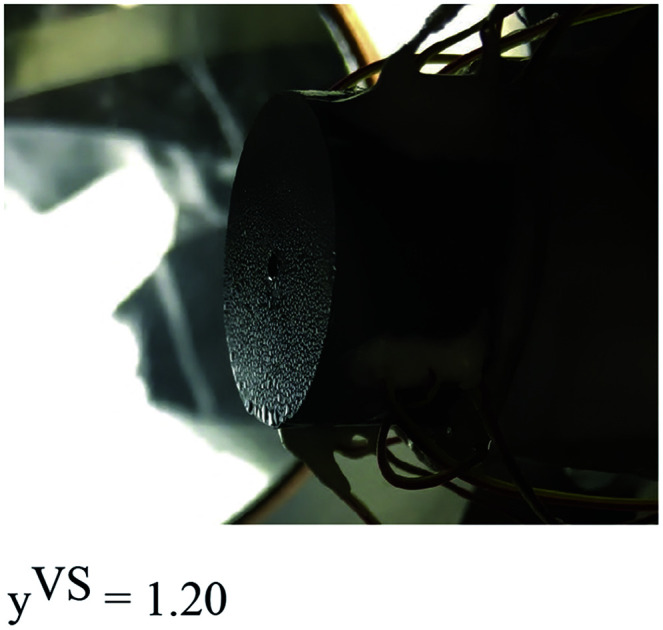

## 1 Introduction

Dropwise condensation has been studied widely in the literature. This process is observed in many natural processes, including condensation on lotus leaves and butterfly wings^1–3^ or industrial processes such as phase change heat transfer applications.^4^ In comparison to filmwise condensation, dropwise condensation has been of great interest because of its higher rate of energy transport.^5^ Due to the enhancement of the energy transport in dropwise condensation, a wide range of research has been performed to investigate the mechanism of dropwise condensation^6–9^ and to develop engineered surfaces that induce dropwise condensation mode.^4^ To investigate the mechanism of dropwise condensation, the concept of molecular clustering has been applied and has been studied extensively.^3,10,11^ Despite the studies that have been performed on this subject, a physical understanding of the initiation of dropwise condensation is still not complete.^3,12^

Recently, the zeta adsorption isotherm (ZAI) theory^13^ was applied to investigate the role of molecular clustering under thermal disequilibrium conditions on the initiation of filmwise condensation for a system of heptane vapour adsorbing on a Si surface.^14^ For the described system, it was experimentally found that the contact angle does not form on the Si surface, and the liquid heptane fully wets the solid surface.^8,14^ The experimental results of the initiation of filmwise condensation was examined using the ZAI theory.

In this work, the initiation of condensation for two systems of vapours adsorbing on a Si surface is investigated: toluene and octane vapours. Experimental measurements of the equilibrium amount of vapour adsorbed on Si are presented, and the measurements of the initiation of the liquid phase are performed to determine condensation mode of these two vapours. Furthermore, the ZAI theory, which is based on the assumption of the formation of molecular clusters in the adsorbate, is applied, and the conditions for the formation of liquid droplets and the liquid film are investigated.

## 2 Background

### 2.1 The amount adsorbed, *η*_td_, under the steady, thermal disequilibrium conditions

The equilibrium ZAI theory^13^ predicts the amount of vapour adsorbed on a solid surface under the equilibrium condition in terms of the pressure ratio, *x*^V^, and the isotherm constants (*M*, *c*, *α*, *ζ*_th_). The theory has been shown experimentally to correctly predicts the amount of vapour adsorbed at the equilibrium condition.^13,15–21^

In a recent study,^14,22^ this theory has been extended to the steady, thermal disequilibrium conditions where the temperature discontinuity exists at the solid–vapour interface.^23–26^ At these conditions, a vapour at a temperature, *T*^V^, is exposed to a solid surface at a lower temperature, *T*^S^, and a temperature function, *y*^VS^, is defined as,1
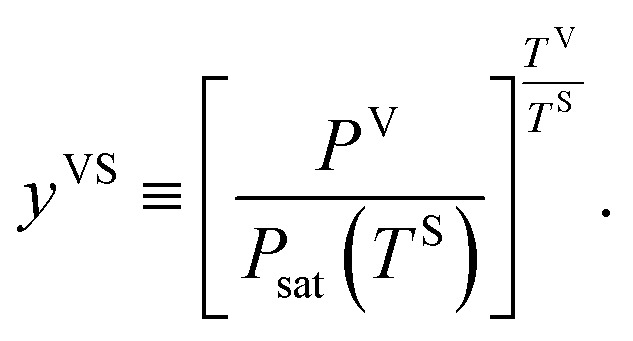


It is hypothesized that the adsorbate, which is at local equilibrium at the solid temperature, *T*^S^, consists of molecular clusters. Each molecular clusters can contain *ζ* molecules where *ζ* can be 1,2,3,… *ζ*_m_. The variable *ζ*_m_ is the maximum number of molecules in a cluster which has been shown to depend on the potential energy of the adsorbate.^14^ Since the adsorbate is at local equilibrium, the chemical potential of the molecules in the vapour phase, *μ*^V^, has the same value as the chemical potential of the adsorbed clusters with one molecule. Consequently, the one molecule clusters are allowed to be exchanged with the molecules in the vapour phase.^22^ The adsorbed molecular clusters on the solid surface, are formed as a result of the interaction of the one-molecule clusters and the multiple-molecule clusters.

Further, the molecular clusters are approximated as harmonic oscillators with the fundamental frequencies that depend on the number of molecules in the clusters, and the canonical ensemble and statistical thermodynamics are applied to construct an expression for the chemical potential of a cluster with *ζ* molecules. Two isotherm parameters, *β*_td_ and *c*_td_ are defined and an expression for the number of empty adsorption sites, *a*_0_, and the number of clusters with *ζ* molecules, *a*_*ζ*_, are developed as expressed below,2
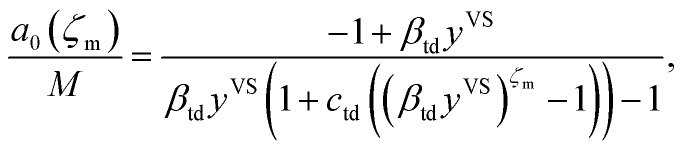
and3
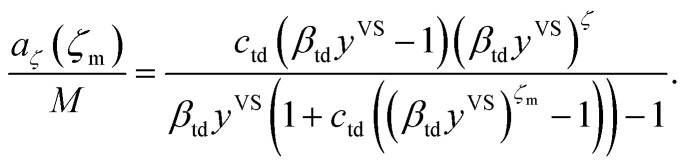


The expression for the total number of molecules adsorbed, *η*_td_, is constructed by summing the number of molecules in each cluster type, *ζa*_*ζ*_, over the possible values of *ζ*, *i.e.* from 1 to *ζ*_m_^14,22^4
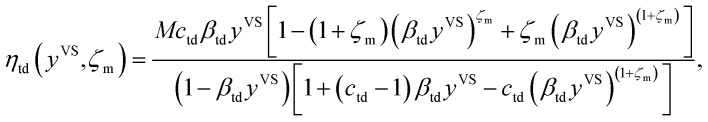
where *M* is the number of adsorption sites per unit area, *β*_td_ and *c*_td_ are the isotherm constants.

In the thermal equilibrium limit when *T*^S^ and *T*^V^ have the same value and the vapour phase pressure, *P*^V^, is less than the saturation vapour pressure corresponding to this temperature, *y*^VS^ becomes *x*^V^:5
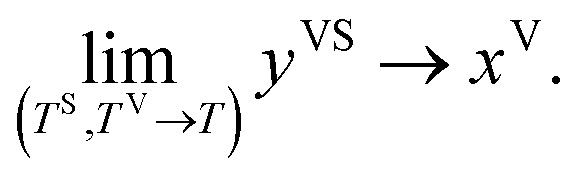


It is shown that in this limit, the isotherm constants, *β*_td_(*T*^V^, *T*^S^) and *c*_td_(*T*^S^) reduce to the equilibrium zeta adsorption isotherm constants, *α*(*T*) and *c*(*T*).

Finally, in this limit *η*_td_(*y*^VS^) reduces to *n*^SV^(*x*^V^), the amount adsorbed under equilibrium condition using the equilibrium ZAI theory:^22^6
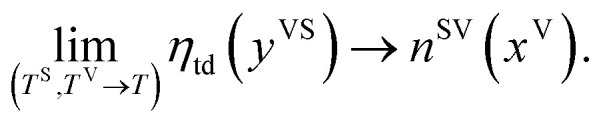


### 2.2 Surface tension of the solid–vapour interface

When the solid surface is in equilibrium with the vapour phase and *y*^VS^ ≤ 1, the Gibbs adsorption equation is denoted,^13,27,28^

7 d*γ*^SV^ = −*n*^SV^d*μ*^SV^.

If the vapour phase is approximated as an ideal gas, eqn (7) can be written as8
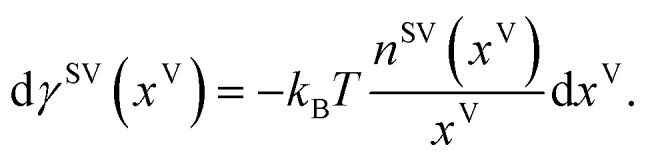


Under the thermal disequilibrium conditions, when the solid temperature is reduced below the vapour temperature, and *y*^VS^ > 1, thermodynamics cannot be applied. Therefore, the method proposed in the previous study^14^ is applied to construct an expression for d*γ*^SV^(*y*^VS^),9
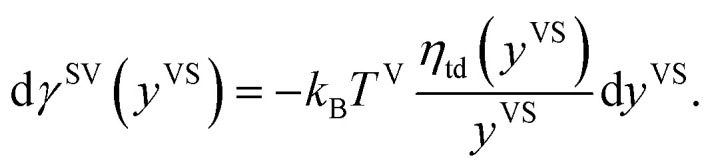


It is found that in the limit of *y*^VS^ approaching *x*^V^, eqn (9) is reduced to eqn (8). The surface tension of the solid–vapour interface, *γ*^SV^, is obtained by integrating eqn (9) from zero to an arbitrary value of *y*^VS^ less than *y*^VS^_w_. Therefore, an expression is developed for *γ*^SV^ in terms of *y*^VS^, the isotherm constants and an integration constant, *C*. Also, it is known that *γ*^SV^(*y*^VS^, *ζ*_m_) at *y*^VS^ equals zero is equal to the surface tension of the solid in the absence of adsorption, *γ*^S0^. Therefore, the expression developed for *γ*^SV^ is written as,10
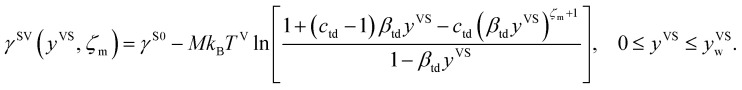


It is hypothesized that at *y*^VS^_w_, *γ*^SV^(*y*^VS^_w_) reduces to the surface tension of the liquid–vapour interface, *γ*^LV^. Therefore, the expression for *γ*^S0^ is denoted as11
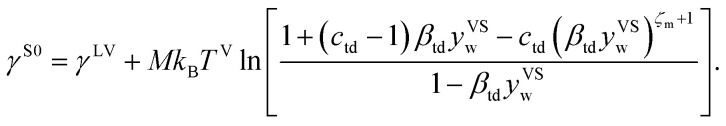


The value of *γ*^S0^ for Si was determined from the measurement of heptane vapour adsorbing on a Si surface and was reported to be 128.4 ± 3 mJ m^−2^.^14^

## 3 Experimental measurements of toluene vapour adsorbing on Si

Two sets of measurements were performed in this study: the measurements in the equilibrium range when *y*^VS^ ≤ 1 and the measurements in the thermal disequilibrium range when *y*^VS^ > 1.

### 3.1 Measurements in the equilibrium range

**Fig. 1:**
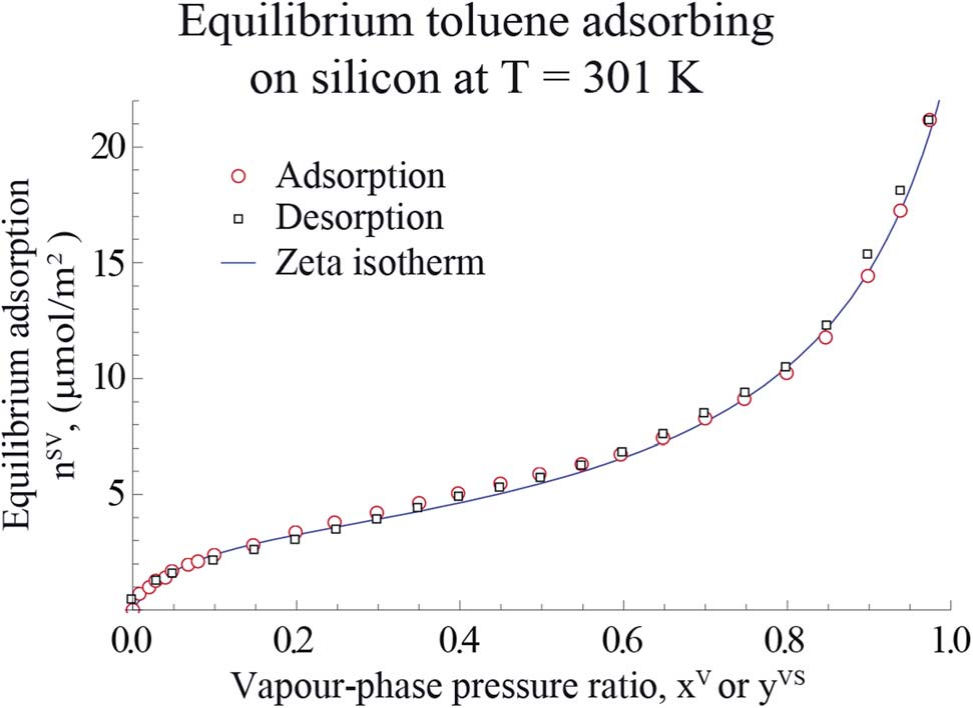
The measured amount of toluene vapour adsorbing-on and desorbing-from a silicon nano powder and the predicted amount adsorbed using the expression developed, *η*_td_ (eqn (4)), are illustrated in this figure. The data points are used with eqn (4) to determine the values of the isotherm constants: *M*, *c*_td_ and *β*_td_ and the threshold value of the number of molecules in a cluster, *ζ*_th_. The error bars of the measured amount adsorbed are within the symbols.

For the measurements in the equilibrium range, the equilibrium gravimetric measurements of adsorption and desorption isotherms of toluene vapour adsorbing on silicon nanopowder (Sigma Aldrich) was used. The same measurement procedure as that described before^18–20,22^ was applied. A sample of approximately 10 mg of the silicon nanopowder was placed in the microbalance of an adsorption instrument (Surface Measurement Systems, DVS Advantage) and heated to 423 K with dry *N*_2_, and held at this condition for 4 hours. Afterwards, the sample was cooled to 301 K, and the gravimetric measurements were made of the amount adsorbed, *n*^SV^_g_ (±0.5 μg), when the sample was exposed to anhydrous, toluene (Sigma Aldrich, 99% purity) for the range of the pressure ratio, 0 < *x*^V^ ≤ 0.97. It is assumed that the system was reached the equilibrium condition when the change in the amount adsorbed was less than 0.02% in 20 minutes at a value of *x*^V^. Following the method described by Zandavi and Ward,^19^ the amount adsorbed per unit mass, *n*^SV^_g_, was converted to the amount adsorbed per unit area, *n*^SV^, using the specific surface area, A_s_(Si)^22^ which is shown in Fig. 1.

### 3.2 Measurements in the non-equilibrium range

For the measurements in the steady, thermal disequilibrium conditions, the same procedure was used that was described in details in the study by Yaghoubian *et al.*^14,22^ Briefly, the experimental apparatus used in this study consists of a vacuum chamber that has four viewports and a silicon substrate, which was mounted on a stainless-steel cylinder. A cooling fluid was circulated in the stainless-steel cylinder in order to keep the temperature of the silicon substrate at a constant value, *T*^S^. The stainless-steel chamber was also thermostated at a constant temperature, *T*^V^.

One side of the Si disk, which was polished to a surface roughness of 1 nm (Sil'tronix S.T., Fr), and it could be viewed by the light source of a UV-visible interferometer (Filmetrics, USA). In order to measure the solid temperature, 12 thermocouples were mounted in the silicon substrate, four at every three longitudinal depths. At each depths, thermocouples were placed at the three radial positions from the centre line of the silicon substrate, one at each 90° rotation around the disk.

Before running an experiment, the chamber was evacuated to a pressure of 10^−6^ Pa using a stainless-steel turbo-molecular diffusion pump and held at this condition for 48 hours. Then, a sample of the gas–vapour mixture in the chamber was analyzed with a residual gas analyzer (SRS Model RGA 200). The composition of the gas–vapour mixture existing in the chamber before running the experiment was found to be *N*_2_. The toluene vapour that was used in the experiments was first degassed, and then the vapour phase pressure and the temperature in the degassing flask were measured. The vapour phase pressure corresponded to the saturation vapour pressure at the measured temperature. Then, the toluene vapour was introduced into the chamber, and the system brought to the steady state condition^22^ at the values of the temperature function greater than unity.

### 3.3 Determination of the isotherm parameters

As explained in Section 2.1, when *y*^VS^ which is defined in eqn (1) is less than unity, thermal equilibrium exists in the system. In the thermal equilibrium limit, the amount adsorbed under thermal disequilibrium conditions, *η*_td_, is reduced to the equilibrium amount adsorbed, *n*^SV^. Therefore, the equilibrium adsorption measurements of toluene on a Si nanopowder and the nonlinear regression package of Mathematica™ were applied to determine the values of the equilibrium isotherm constants, *M*, *c*, *α*, *ζ*_th_.^18–20,22^

As demonstrated by Ward and Wu,^13^*ζ*_th_ gives the best agreement of the predicted amount adsorbed with the measurements under the equilibrium condition. For the values of *ζ* greater than *ζ*_th_ in the equilibrium range, the agreement between the predicted and the measured amount adsorbed does not increase further. The maximum number of molecules in a cluster, *ζ*_m_, should be determined from the measurements under the thermal disequilibrium conditions. In the thermal disequilibrium range, *y*^VS^ > 1 and the solid temperature is reduced below the vapour phase temperature; however, the effect of reducing the solid temperature on the isotherm constants is assumed to be negligible. Therefore, the values of the isotherm parameters, *M*, *c*_td_ and *β*_td_ are approximated from the measurements in the equilibrium range and are shown in Table 1.

Equilibrium zeta adsorption isotherm constants at 301 K, and thermal disequilibrium parameters for toluene adsorbing on silicon

**Table:** 

Equilibrium	*M*_g_ μmol mg^−1^	*σ̄*_toluene_ (Å)^2^	*M* μmol m^−2^	*c*	*α*	*ζ*_th_
0 < *x*^V^ ≤ 0.97	0.053 ± 0.001	50 ± 2	3.32 ± 0.10	20.5 ± 2.3	0.862 ± 0.005	90

**Table:** 

Thermal dis-equilibrium	*M*_g_ μmol mg^−1^	*σ̄*_toluene_ (Å)^2^	*M* μmol m^−2^	*c*_td_(*T*^S^)	*β*_td_(*T*^V^, *T*^S^)	*ζ*_m_
1 < *y*^VS^ < 1.30	0.053 ± 0.001	50 ± 2	3.32 ± 0.10	20.5 ± 2.3	0.862 ± 0.005	213

The gravimetric adsorption and desorption measurements of toluene vapour adsorbing on Si and the predicted amount adsorbed calculated from the expression developed in Section 2.1, *η*_td_, are depicted in Fig. 1.

**Fig. 2:**
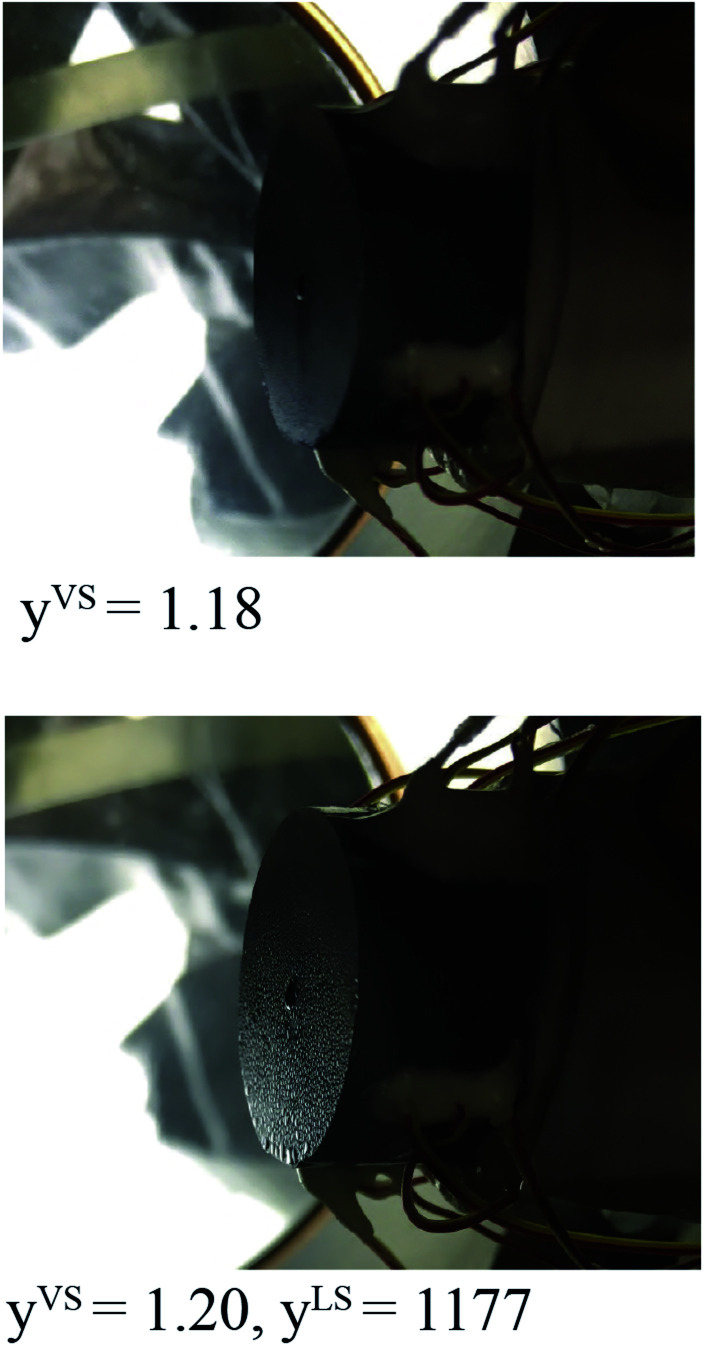
When *y*^VS^ was set at 1.18, no phase change was observed on the silicon surface; however, when *y*^VS^ was increased to 1.20, the liquid droplets become discernible on the surface.

The value of *ζ*_m_ is determined from the experimental measurements in the thermal disequilibrium range. The surface of the Si substrate exposed to the toluene vapour is photographed at *y*^VS^ equals 1.18 ± 0.01 in a steady state condition and is depicted in Fig. 2. The phase change has not been started, and no droplet is observed on the Si surface. When *y*^VS^ is increased to 1.20 ± 0.01, it is observed that the phase change has been started, and the toluene droplets become discernible on the Si substrate. It is observed that once *y*^VS^ is increased above 1.20, droplets grow and start to drain from the Si surface. We assume that at *y*^VS^ equals 1.19 ± 0.01, the liquid phase is formed on the Si surface. This condition is defined as the wetting condition, and the temperature function of wetting is *y*^VS^_w_.

Further, it is hypothesized that at wetting, the surface tension of the solid–vapour interface, *γ*^SV^(*y*^VS^_w_), is equal to the surface tension of the liquid–vapour interface, *γ*^LV^. In the expression developed for *γ*^SV^(*y*^VS^_w_) (eqn (12)), the values of the isotherm parameters, the surface tension of the solid in the absence of adsorption, and the temperature function of wetting is known. Therefore, eqn (12), is solved for *ζ*_m_, and the value of *ζ*_m_ is found to be 213.12
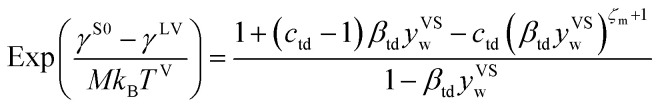


The values of the isotherm constants in the thermal disequilibrium range and the maximum number of molecules adsorbed in a cluster are listed in Table 1. The value of *γ*^S0^ used to solve eqn (12) was reported to be 128.4 ± 3 mJ m^−2^.^14^

## 4 Calculating the critical size of liquid toluene droplets

As described in Section 3.3, *y*^VS^_w_ is defined as the condition at which the liquid phase is formed. For *y*^VS^ greater than *y*^VS^_w_, the condensed droplets are observed on the Si surface. The possible explanation of this observation has been given in terms of the distribution of the clusters in the adsorbate. The distribution of the clusters for the system of toluene vapour adsorbing on the Si surface is determined and is depicted in Fig. 4. For each value of *y*^VS^, the critical number of molecules in the adsorbate is defined as *ζ*_c_. The results demonstrate that the number of clusters with more than *ζ*_c_ molecules increases, while the number of clusters with the number of molecules less than *ζ*_c_ decreases.

We suggest that *ζ*_c_ determines the critical size of the clusters. The clusters larger than the critical size clusters, which form nanoscale droplets, grow by condensation,^3,12^ while smaller clusters evaporate from the Si surface.

### 4.1 Determination of the critical size of clusters

To determine the value of *ζ*_c_, the expression developed for *a*_*ζ*_ which is also given in eqn (3) is used. We also impose a condition that the critical size of a cluster is determined where the partial derivative of *a*_*ζ*_ with respect to *y*^VS^ becomes zero. In other words, for a certain *y*^VS^ the critical size of a cluster, *ζ*_c_, is calculated where the *a*_*ζ*_ curve has an extremum *i.e.* the *a*_*ζ*_ curves with *ζ* greater than *ζ*_c_ have a positive slope and grow while the curves with *ζ* less than *ζ*_c_ have a negative slope and diminish. This condition is expressed as,13
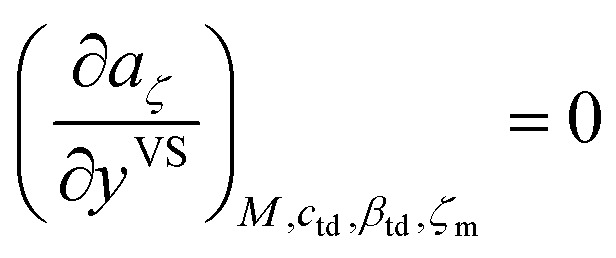


**Fig. 3:**
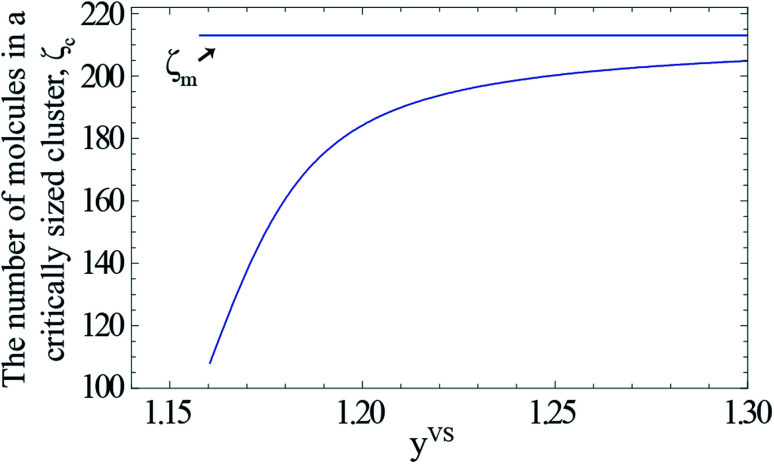
The calculated values of the number of molecules in a critically sized cluster, *ζ*_c_, is indicated by the solid curve. The value of *ζ*_m_ (213) is indicated by the horizontal solid line. The range of the possible cluster types which are growing at each value of *y*^VS^ is, *ζ*_m_ − *ζ*_c_(*y*^VS^).

**Fig. 4:**
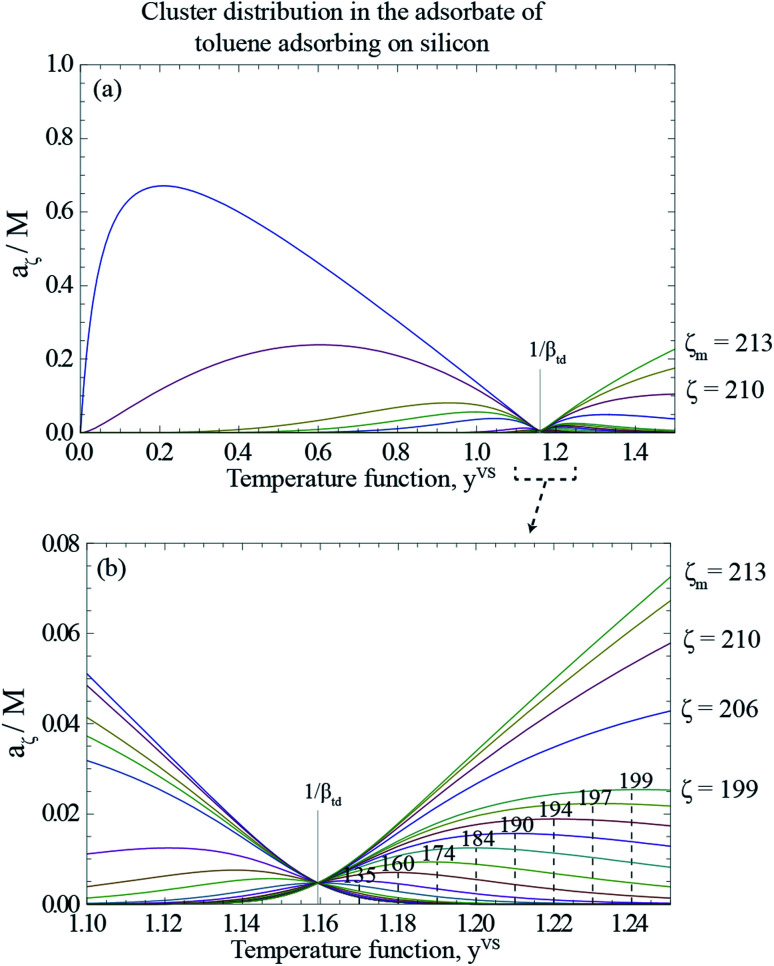
(a) The calculated cluster distribution of toluene vapour adsorbing on silicon as a function of the temperature function, *y*^VS^, using eqn (3), and the parameters listed in Table 1. (b) The calculated cluster distribution for the range of *y*^VS^ from 1.10 to 1.24. At *y*^VS^ → 1/*β*_td_, the concentration of each cluster type is the same, but those with a *ζ* less than *ζ*_c_(1/*β*_td_), are found to have a negative slope at *y*^VS^(1/*β*_td_), *i.e.*, to be evaporating; while those with a *ζ* greater than *ζ*_c_(1/*β*_td_) have a positive slope and are growing by condensation.

For a given value of *y*^VS^, a numerical procedure is used to solve eqn (13), using the expression developed for *a*_*ζ*_, eqn (3). The calculated values of *ζ*_c_ at each *y*^VS^ are shown in Fig. 3. Note that at each *y*^VS^, those clusters with more than *ζ*_c_ molecules are expected to grow by condensation, but the clusters with the fewer number of molecules are expected to evaporate. The cluster distribution developed in eqn (3) is also shown in Fig. 4. It is demonstrated in this figure that when *β*_td_*y*^VS^ is approaching unity, the concentration of each cluster type is the same. When *y*^VS^ is increased further, clusters with the larger number of molecules grow in number and become the main cluster type in the adsorbate.

### 4.2 The critical size droplets

For the range of *y*^VS^ greater than *y*^VS^_w_ where droplets exist on the Si surface, the pressure ratio in the liquid phase at the three-phase line, *y*^LS^, is defined,14
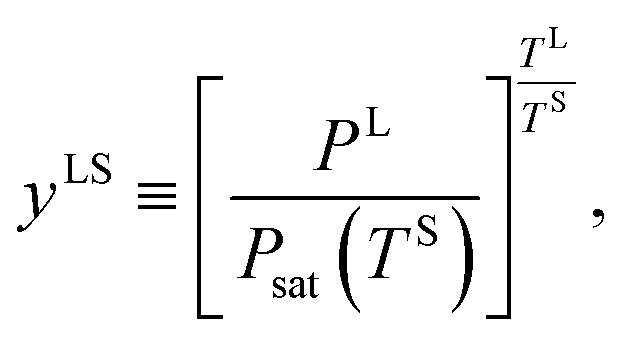
where *P*^L^ is the liquid pressure at the three-phase line, and *T*^L^, *T*^S^ are the liquid temperature and the solid temperature, respectively. Corresponding to each subcooling, the critical size of a cluster, *ζ*_c_, is determined. It is predicted that as the subcooling increases, condensation occurs on the clusters with the number of molecules larger than *ζ*_c_, and these clusters grow and form nano scale droplets, while the clusters with the number of molecules less than *ζ*_c_ evaporate and disappear from the Si surface. It is assumed that a critical size droplet with a radius of *R*_d_ contains *ζ*_c_ molecules, and a method is proposed to calculate the contact angle, *θ*_c_, of these droplets, which is explained below.

When a liquid droplet forms on the silicon surface, the pressure in the liquid is greater than the pressure in the vapour phase. Therefore, the pressure ratios, *y*^LS^, eqn (14), and *y*^VS^, eqn (1), are defined. In order to determine the relation between *y*^LS^ and *y*^VS^, a series of assumptions have been made. First, it is assumed that the temperature of the liquid phase of the initial droplets which are formed on the solid surface is *T*^S^. The solid temperature corresponding to the temperature function of wetting is denoted *T*^S^_w_. Therefore, the condition for the existence of the liquid phase is that *T*^S^ < *T*^S^_w_. If this condition is applied to the definition of *y*^LS^, it will reduce to:15
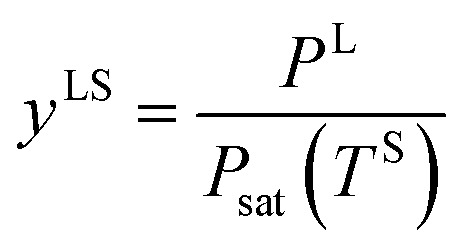


Second, it is assumed that the chemical potential of the liquid, vapour, solid–vapour and solid–liquid phases are equal at the three-phase line which is also shown below,

16 *μ*^V^ = *μ*^L^, *μ*^SV^ = *μ*^V^, *μ*^SL^ = *μ*^L^

where V, L, SV and SL are the vapour phase, liquid phase, solid–vapour interface and solid–liquid interface, respectively. If the molar specific volume of the liquid at saturation is denoted as *v*_f_, and the isothermal compressibility as *k*_T_, then provided |*k*_T_[*P*_sat_(*T*^S^) − *P*^L^]| ≪ 1, the chemical potential of the liquid phase, *μ*^L^(*T*^S^, *P*^L^), is written as

17 *μ*^L^(*T*^S^, *P*^L^) = *v*_f_(*P*^L^ − *P*_sat_(*T*^S^)) + *μ*^L^(*T*^S^, *P*_sat_(*T*^S^))

The vapour phase is also approximated as an ideal gas. Therefore, *μ*^V^(*T*^V^, *P*^V^), is written in the following form ref. 2218
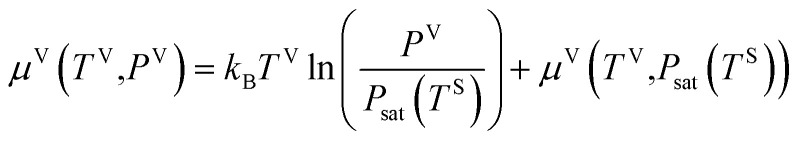
where *k*_B_ denotes the Boltzmann constant. When eqn (16) to (18) are combined, the expression for the liquid phase pressure is developed19
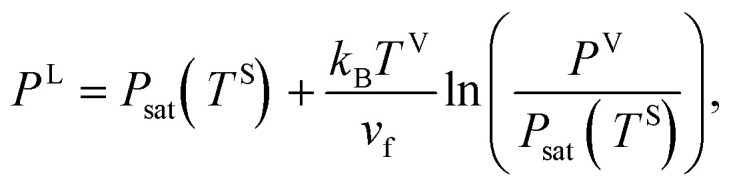
which can also be written as20
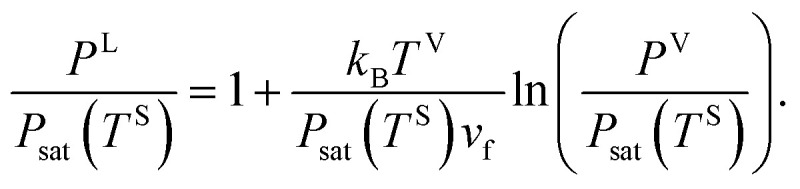


Further,21
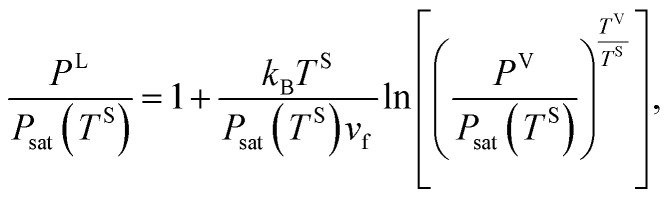
where it is shown that *P*^V^ is equal to *P*_sat_(*T*^V^). If eqn (1) and (14) are substituted in eqn (21), the result can be written as,22
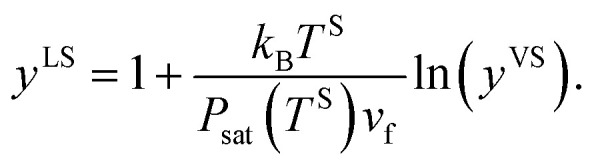


If the molar specific volume of the gas phase is *v*_g_ = *k*_B_*T*^S^/*P*_sat_(*T*^S^), eqn (22) is simplified to,23
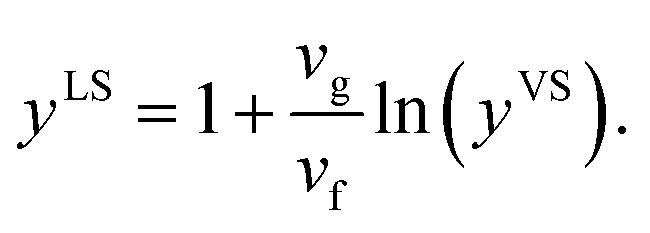


Third, it is assumed that the Laplace equation would be valid at the liquid–vapour interface. Therefore, it is written as,

24 *P*^L^ − *P*^V^ = *γ*^LV^(*C*^LV^_1_ + *C*^LV^_2_),

where the surface tension of the liquid–vapour interface at the solid temperature, *T*^S^, is denoted as *γ*^LV^ and *C*^LV^_*i*_ is one of its curvatures at the liquid–vapour interface. The initial droplets, that are formed on the solid surface, are assumed to be spherical, with the uniform *curvature at the liquid–vapour interface*. Therefore,25
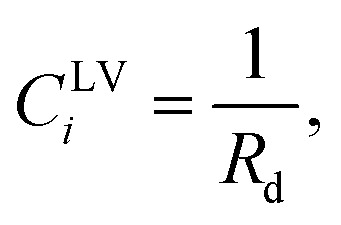
where *R*_d_ is the radius of the liquid–vapour interface of a critical size droplet. If eqn (25) is substituted in eqn (24), the result can be written in the following form,26
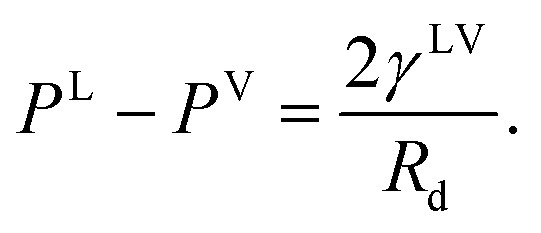


Therefore, the radius of the liquid–vapour interface of a critical size droplet, *R*_d_, is obtained by combining eqn (26) and (19),27
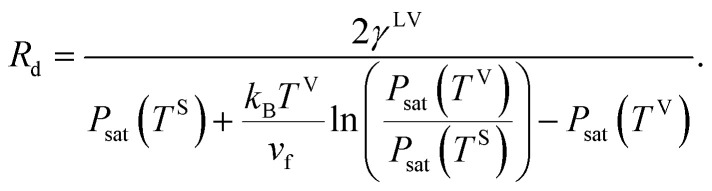


The liquid droplet is approximated to be spherical with the liquid–vapour interface of *R*_d_ and the contact angle of *θ*_c_. Therefore, the volume of this spherical droplet is determined from the geometry,28
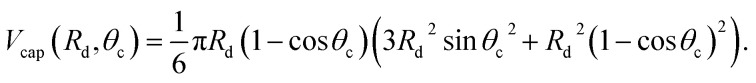


Also, if the number of molecules in the droplet, *ζ*_c_, is known, the volume of the droplet, *V*_c_(*R*_d_, *θ*_c_), can be determined from the expression below,29
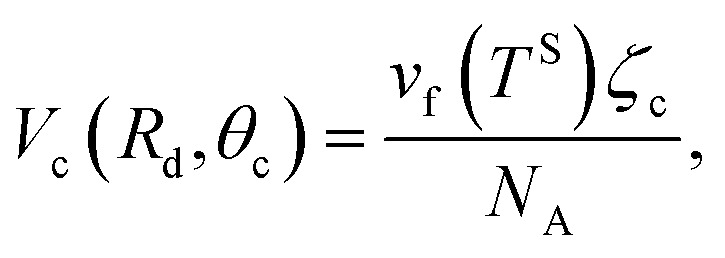
where *N*_A_ denotes the Avogadro number. If one equates eqn (28) and (29), an expression would be developed in terms of *R*_d_ and *θ*_c_,30
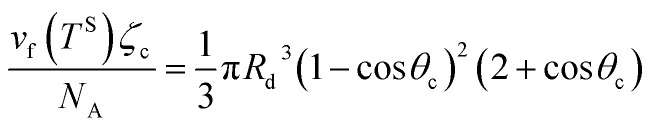


If eqn (27) and (30) are solved numerically, the values of the contact angle, *θ*_c_, is determined as a function of *T*^S^, *i.e. θ*_c_ = *f*_1_(*T*^S^). The pressure ratio in the liquid phase, *y*^LS^ (eqn (14)), is also a function of *T*^S^ (*y*^LS^ = *f*_2_(*T*^S^)); therefore, *T*^S^ is equal to the inverse of the function of *y*^LS^ which can be written as: *T*^S^ = *f*_2_^−1^(*y*^LS^). Therefore, the contact angle, *θ*_c_, can be written as a function of *y*^LS^. That is,

31 *θ*_c_ = *f*_1_(*f*_2_^−1^(*y*^LS^)) = *g*(*y*^LS^)

**Fig. 5:**
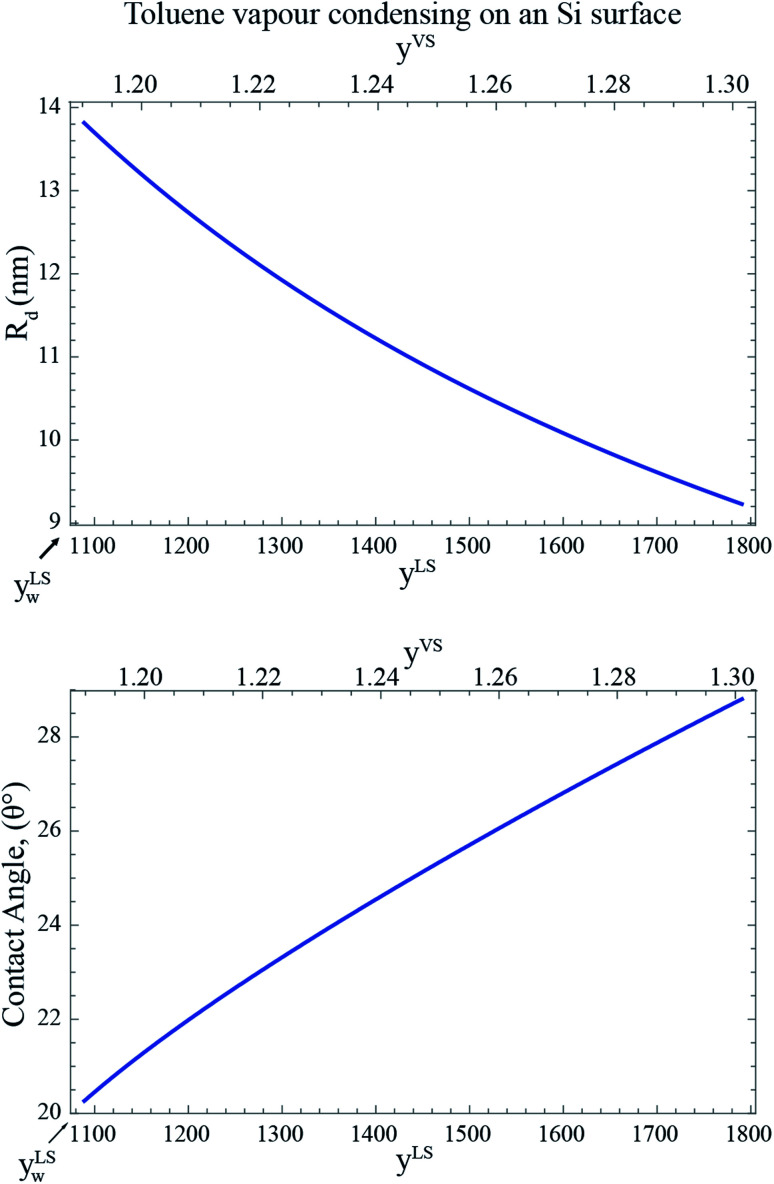
The calculated radius, *R*_d_, and the contact angle, *θ*_c_, of a critical size droplet as a function of the liquid phase pressure ratio, *y*^LS^ are calculated from eqn (27) and (30). It is illustrated in this figure when subcooling increases, the radius of the critical size droplets decreases, while the contact angle increases. It is predicted that the droplets with the radius greater than *R*_d_ grow on the Si surface.

Corresponding to each value of *y*^LS^, the radius of the critical size droplet, *R*_d_, and the contact angle, *θ*_c_, of the critical size droplet, which contains *ζ*_c_ molecules are determined. The results, which are summarized in Fig. 5, suggest that increasing *y*^LS^, decreases the critical radius of droplets, *R*_d_, and increases the contact angle of these droplets, *θ*_c_.

If one compares the results illustrated in Fig. 5 with the distribution of the clusters in Fig. 4, the following conclusion would be obtained. When the subcooling is increased on the surface, the clusters with a larger number of molecules dominate the adsorption sites while the clusters with a smaller number of molecules evaporate and disappear from the surface. These growing clusters with the *ζ*_c_ number of molecules, form nanoscale droplets with the radii of *R*_d_ and the contact angles of *θ*_c_. It is predicted that the droplets with the number of molecules larger than *ζ*_c_ and the radius greater that *R*_d_ starts to grow on the Si surface.^29^ By increasing the surface subcooling, these droplets grow further and form microscale droplets which are photographed and are shown in Fig. 2.

Since the Si surface is placed vertical, the gravitational force is acting on the droplets.^6^ The effect of the gravitational force on the thickness of the adsorbed film was investigated in the recent work by Yaghoubian *et al.*^14^ This force pulls the drop downward while the surface tension forces act in the opposite direction. When the droplets become large enough, that the gravitational force overcomes the surface tension forces, the droplets start to drain from the surface. For toluene vapour condensing on the Si surface, drainage start at *y*^LS^ equals 1177 at the bottom of the Si surface, and when *y*^LS^ is increased to 1462, droplets drain form the entire surface.

## 5 Experimental measurements of the octane vapour adsorbing on Si

**Fig. 6:**
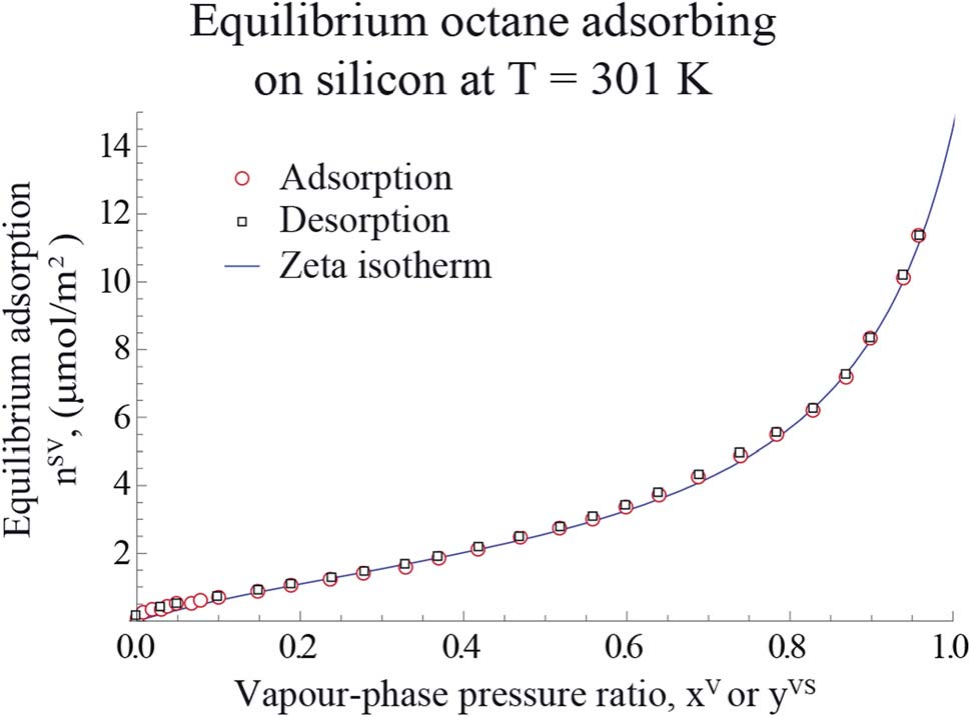
The measured amount of octane vapour adsorbing-on and desorbing from a silicon nano powder are shown with the hollowed circle and rectangles, respectively. The predicted amount of adsorbed vapour on the solid surface is plotted using the expression developed for, *η*_td_ (eqn (4)), and is shown with the blue line. Note that the error bars of the measurements are within the symbols.

Following the procedure described in Section 3, the equilibrium gravimetric measurements of adsorption and desorption isotherms of octane vapour adsorbing on silicon nanopowder were performed and the amount of vapour per unit mass of solid, *n*^SV^_g_, was measured. By applying the specific surface area of Si,^19^*n*^SV^_g_ was converted to the amount of vapour adsorbed per unit area of the solid, *n*^SV^. The experimental measurements are illustrated in Fig. 6.

**Fig. 7:**
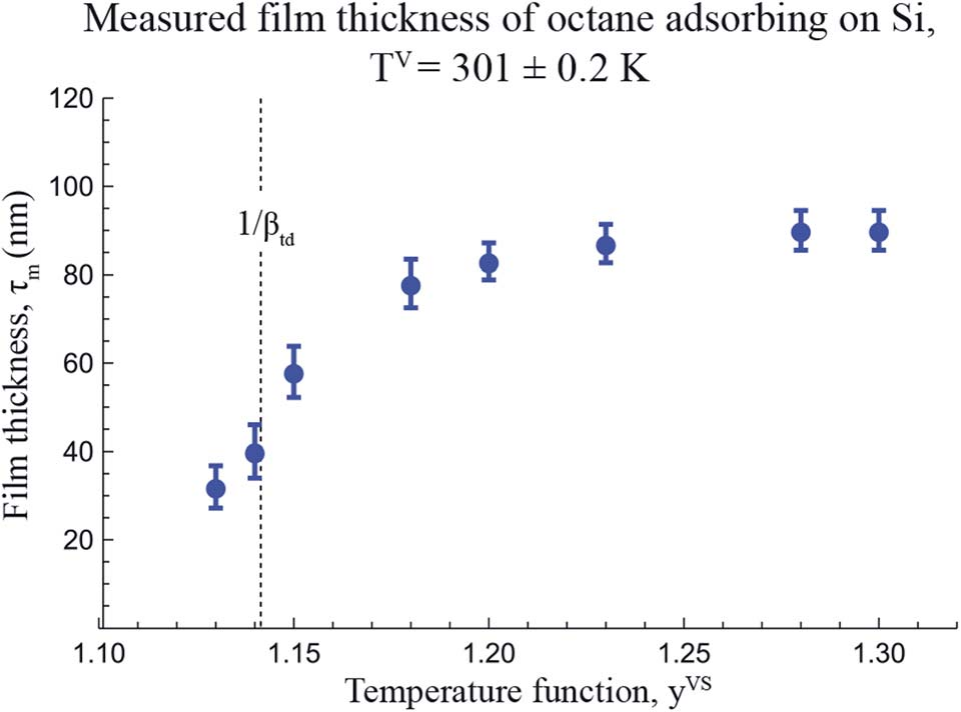
The measured thickness of adsorbate for a system of octane vapour adsorbing on an Si surface as a function of temperature function, *y*^VS^, at *z* = 14.4 mm are illustrated in this figure. The error bars indicates the error in measuring the thickness determined from at least ten repeated measurements using the software provided by the manufacturer.

Using the equilibrium measurements, the values of the isotherm parameters, *M*, *c*_td_, *β*_td_ and *ζ*_th_ are determined and are listed in Table 2. To determine the maximum number of molecules in a cluster, the thickness of the octane adsorbate was measured at *z* = 14.4 mm from the bottom of the Si substrate. The experimental procedure that was used to make the measurements of the thickness of the adsorbed film was explained in detail in a recent study by Yaghoubian *et al.*^14,22^ The experimental measurements are depicted in Fig. 7. As illustrated in this figure, when *y*^VS^ is increased above 1.18, the thickness of the adsorbate reaches a plateau value. It is assumed that in this range, the adsorbate can be approximated as a liquid phase; therefore, the specific volume of the adsorbed fluid may be approximated as that of a saturated liquid.^22^ Using this assumption, the measurements of the film thickness were converted to the amount adsorbed, and the zeta adsorption isotherm was applied to determine the value of the maximum number of molecules in a cluster, *ζ*_m_. The calculated value of *ζ*_m_ was found to be 299, which is also listed in Table 2.

Equilibrium zeta adsorption isotherm constants at 301 K, and the thermal disequilibrium parameters of the octane adsorbing on silicon

**Table:** 

Equilibrium	*M*_g_ μmol mg^−1^	*σ̄*_octane_ (Å)^2^	*M* μmol m^−2^	*c*	*α*	*ζ*_th_
0 < *x*^V^ < 0.96	0.039 ± 0.001	89 ± 4	1.87 ± 0.10	4.4 ± 0.4	0.876 ± 0.005	80

**Table:** 

Thermal dis-equilibrium	*M*_g_ μmol mg^−1^	*σ̄*_octane_ (Å)^2^	*M* μmol m^−2^	*c*_td_(*T*^S^)	*β*_td_(*T*^V^, *T*^S^)	*ζ*_m_
1 < *y*^VS^ < 1.30	0.039 ± 0.001	89 ± 4	1.87 ± 0.10	4.4 ± 0.4	0.876 ± 0.005	299

**Fig. 8:**
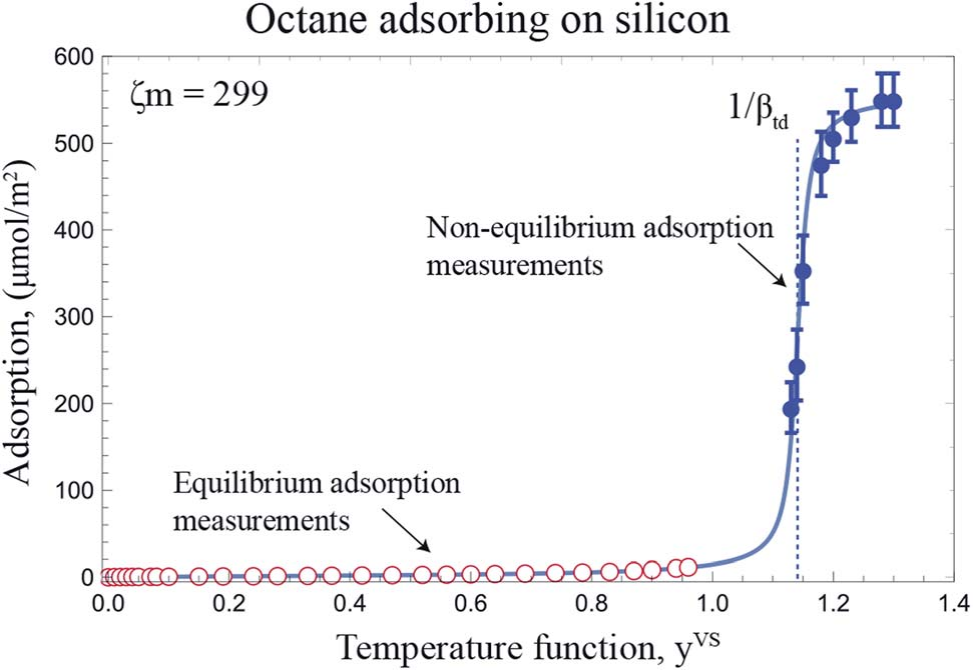
The adsorption of octane vapour on silicon is depicted for the equilibrium and non-equilibrium range of *y*^VS^ at *z* = 14.4 mm. For *y*^VS^ ≥ 1, the calculated amount of vapour adsorbed using eqn (4) and the isotherm constants listed in Table 2 is shown with the solid line.

The measurements of the amount adsorbed in the equilibrium and non-equilibrium range of *y*^VS^ and the predicted amount of vapour adsorbed on as Si surface using the eqn (4) and the calculated isotherm constants listed in Table 2 are illustrated in Fig. 8. As shown in this figure, there is no measurable disagreement between the calculations of the amount adsorbed or either the equilibrium or thermal disequilibrium experimental results. That results support the consistency of the proposed method in the previous study for the filmwise condensation of a vapour on a solid surface.^22^

## 6 Condensation modes of octane, heptane and toluene vapours on a Si surface

So far the experimental measurements of three vapours: octane, heptane and toluene condensing on a Si surface have been illustrated in Section 3, Section 5, and the recent work by Yaghoubian *et al.*^14,22^ The experimental results suggest that the condensation mode of heptane and octane vapours on a Si surface is filmwise, while the condensation mode is dropwise for a toluene vapour condensing on a Si surface. To determine the mechanism that differentiates the condensation modes of these vapours, the surface tension of these vapours are compared which will be discussed further.

### 6.1 Surface tension of the solid–vapour interface

Suppose the lattice of a solid is cleaved to form a surface. Therefore, the atoms near the surface move to a new lattice position which creates a new force field. This force field is what is defined as the surface tension of the solid in the absence of adsorption, γ^S0^. When the solid surface is exposed to a vapour, molecules will be adsorbed on the surface as a collection of molecular clusters which changes the lattice position and reduces the surface tension of the solid–vapour interface, *γ*^SV^, from its value in the absence of adsorption.

**Fig. 9:**
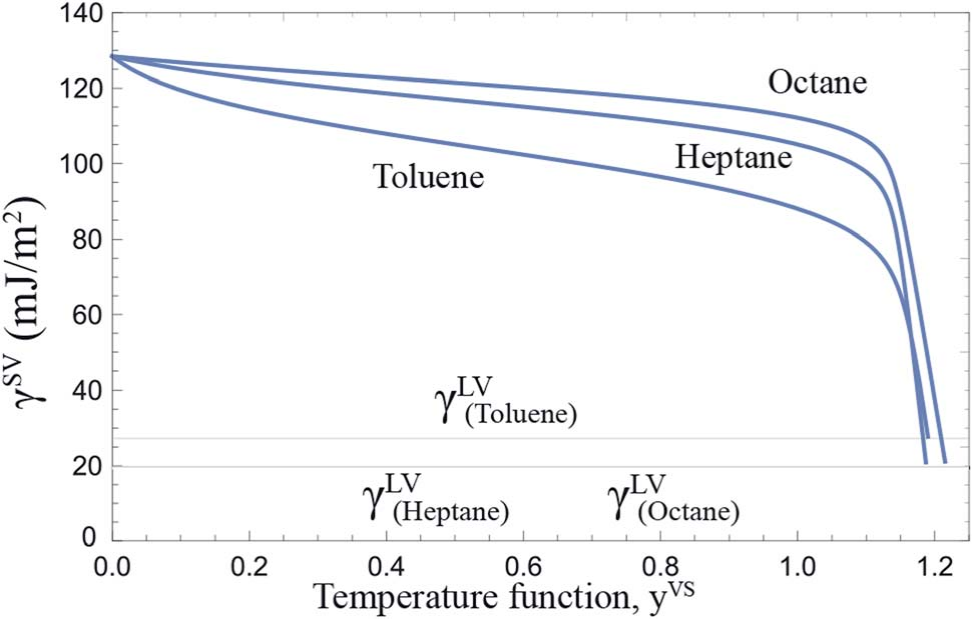
The surface tension of the solid–vapour interface, *γ*^SV^, for a Si surface exposed to octane, heptane and toluene vapours as a function of *y*^VS^ is depicted. The calculated *γ*^SV^ is in the range of 0 ≤ *y*^VS^ ≤ *y*^VS^_w_. The surface tension of Si in the absence of adsorption is 128.4 ± 3 mJ m^−2^. Adsorption lowers the surface tension of solid from its value in the absence of adsorption, *γ*^S0^, to the value of the surface tension of the liquid–vapour interface, *γ*^LV^, at wetting.

As described in Section 2.2, the Gibbs adsorption equation is extended to the thermal disequilibrium conditions and an expression is developed for the surface tension of the solid–vapour interface, *γ*^SV^(*y*^VS^), in terms of the isotherm constants and the maximum number of molecules in a cluster which is expressed in eqn (10). This expression can be applied to the equilibrium and thermal disequilibrium range. The expression for *γ*^SV^ and the isotherm constants of three hydrocarbons: octane, heptane and toluene vapours exposed to the Si surface, listed in Table 3, are applied and the surface tension of the solid–vapour interfaces, *γ*^SV^, are determined for these three vapours. The results are depicted in Fig. 9. It is demonstrated that for all three vapours, adsorption lowers *γ*^SV^ from its value in the absence of adsorption to the surface tension of the liquid–vapour, *γ*^LV^,^30^ at wetting. Furthermore, in the equilibrium range, *γ*^SV^_toluene_ < *γ*^SV^_heptane_ < *γ*^SV^_octane_.

**Table:** Thermal disequilibrium parameters of the zeta adsorption isotherm for three systems of vapours adsorbing on silicon

	*M*_g_ μmol mg^−1^	*σ̄* (Å)^2^	*M* μmol m^−2^	*c*_td_(*T*^S^)	*β*_td_(*T*^V^, *T*^S^)	*ζ*_th_	*ζ*_m_ a
Octane	0.039 ± 0.001	89 ± 4	1.87 ± 0.10	4.4 ± 0.4	0.876 ± 0.005	80	299
Heptane	0.048 ± 0.002	73 ± 3	2.27 ± 0.10	8.5 ± 1.2	0.875 ± 0.005	100	356
Toluene	0.053 ± 0.001	50 ± 2	3.32 ± 0.10	20.5 ± 2.3	0.862 ± 0.005	90	213

a For heptane and octane vapours, *ζ*_m_ is calculated at *z* = 14.4 mm and for toluene vapour *ζ*_m_ is calculated at *z* = 6.5 mm above the bottom of the Si surface.

### 6.2 Investigating the possibility of droplet formation of three vapours on a Si surface

The formation of droplets *vs.* films on the solid surface has been explained in the literature in terms of the surface tensions and the relative surface energy.^3,31^ An experimental study showed that adsorption of hydrocarbons on rare earth oxide ceramics lowers the relative surface free energy and promotes dropwise condensation.^32,33^ It is also found that the fluids with higher surface tensions increase the cohesion interaction between liquid molecules and therefore lower the relative surface free energy.^34^ As illustrated in Table 4, the surface tension of the liquid toluene is 28% higher than *γ*^LV^ of liquid heptane and 24% higher than *γ*^LV^ of liquid octane. Changing the solid surface will also affect the relative surface free energy and therefore may affect the condensation mode.

**Table:** Investigating the possibility of dropwise condensation of three vapours: octane, heptane, toluene on an Si surface

	cos *θ*	γ^SV^(*y*^VS^_w_, *ζ*_th_) mJ m^−2^	*γ* ^SL^(*y*^VS^_w_, *ζ*_m_) mJ m^−2^	*γ* ^LV^ mJ m^−2^
Octane	3.07	85.02	20.88	20.88
Heptane	2.84	75.77	19.72	19.72
Toluene	0.98	54.54	27.55	27.55

We further investigate the possibility of forming liquid droplets on the Si surface for these three vapours by predicting the value of the possible contact angle. As described in Section 2.1, a vapour phase at a temperature *T*^V^ is exposed to a solid surface at a lower temperature, *T*^S^. It is also hypothesized that the adsorbed molecular clusters are at the solid temperature, *T*^S^. Therefore, we assume that at the wetting condition, the clusters which form the liquid phase are still at the solid temperature, *T*^S^, while the vapour phase is at *T*^V^.

When *y*^VS^ is greater than or equal to the wetting condition, *y*^VS^_w_, the adsorbed phase forms the solid–liquid and the solid–vapour interfaces. Since the vapour phase is at the equilibrium temperature, *T*^V^, the values of *ζ*_th_ and the isotherm constants listed in Table 3 are applied to eqn (10) and the surface tensions of the solid–vapour interface, *γ*^SV^(*y*^VS^_w_, *ζ*_th_) are determined. To determine the surface tension of the solid–liquid interface, the expression developed for *γ*^SL^^14^ is applied, and the values of *γ*^SL^(*y*^VS^_s_, *ζ*_m_) are predicted and are demonstrated in Table 4. Finally, the values of cos *θ* are calculated from the Young's equation as expressed below,32
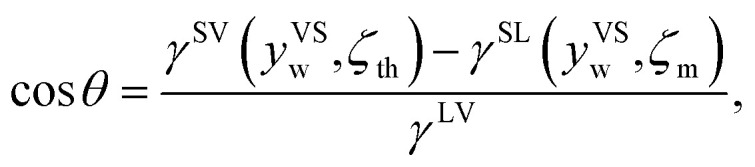
where *γ*^LV^ is the surface tension of the liquid–vapour interface. The results of the calculations, which are shown in Table 4, demonstrate that the predicted value of cos *θ* is less than one for toluene, while it is greater than one for the other two vapours which shows a contradiction; therefore, these results suggest the possibility of dropwise condensation for the system of toluene vapour condensing on a Si surface. This conclusion is consistent with the observation of dropwise condensation for toluene vapour and filmwise condensation of heptane and octane vapours.

## 7 Discussion and conclusion

In this study, initiation of condensation is investigated for two systems of toluene and octane vapours adsorbing on a Si surface. The experimental observations demonstrate that the condensation mode of a toluene vapour on a Si surface is dropwise while an octane vapour condenses on a Si surface as a liquid film.

The ZAI theory and its extension^13,22^ are applied to calculate the amount of vapour adsorbed under equilibrium and thermal disequilibrium conditions. The expression that has been constructed to predict the surface tension of the solid vapour interface, *γ*^SV^,^14^ is applied and the surface tensions of the solid–vapour interface are compared for the octane, heptane and toluene vapours. The calculations demonstrate that adsorption affects *γ*^SV^ and the wetting condition, while this effect has been neglected in the earlier studies.^35,36^ By determining *γ*^SV^ and *γ*^SL^ at wetting, the conditions for the formation of droplets are theoretically investigated. It is predicted that for a toluene liquid on a Si surface, the contact angle greater than zero can form, while calculations suggest that the formation of the contact angle greater than zero is not possible for the heptane and the octane vapours condensing on Si. These predictions are found to be consistent with the experimental measurements.

Furthermore, the condition for the growth of the nanoscale droplets for liquid toluene on a Si surface is investigated. Previous study of the nucleation of a bubble in a liquid–gas solution^29,37–39^ demonstrates that a bubble of the critical size immersed in a liquid–gas solution is in an unstable equilibrium state. It is found theoretically and experimentally that a bubble of a radius slightly greater than the critical size bubble would grow, while under the same conditions, a bubble slightly smaller than this size would dissolve. A similar analysis has been applied here to determine the critical radius of the condensed droplets, *R*_d_. In addition, the critical number of molecules in a cluster, *ζ*_c_, is determined from the cluster distribution constructed in the derivation of the ZAI theory. By combing the expressions developed for *R*_d_ and *ζ*_c_, the contact angle of a critical size droplet, *θ*_c_, is calculated which is depicted in Fig. 5. The calculations, which are illustrated in Fig. 4 and 5, suggest that a droplet with a radius slightly greater than *R*_d_ and the number of molecules larger than *ζ*_c_ grow on the solid surface by condensation, while a droplet of a slightly smaller size would evaporate from the surface.

## Conflicts of interest

There are no conflicts to declare.

## Appendix

### Nomenclature

#### Latin

*a*_0_
: Number of empty adsorption sites
*a*_*ζ*_
: Number of clusters with *ζ* molecules
*C* ^LV^_*i*_
: A curvature at the liquid–vapour interface, *i* = 1 or 2
*c*_td_
: Zeta adsorption isotherm constant, thermal disequilibrium conditions
*c*
: Zeta adsorption isotherm constant
*M*
: Number of adsorption sites per unit area
*n* ^SV^
: Vapour adsorption per unit area of a solid, equilibrium condition, nonporous
*n* ^SV^_g_
: Vapour adsorption per unit weight of a solid, equilibrium condition, nonporous
*P*
: Pressure
*P*_sat_
: Saturation–vapour pressure
*R*_d_
: Radius of a critical size droplet
*T*
: Temperature
*v*_f_
: Specific volume of the liquid at saturation
*v*_g_
: Specific volume of the vapour at saturation
*x* ^V^
: The vapour phase pressure ratio
*y* ^LS^
: The liquid phase pressure ratio
*y* ^VS^
: Temperature function
ZAI: Zeta adsorption isotherm

#### Greek

α
: Zeta adsorption isotherm constant
*β*_td_
: Zeta adsorption isotherm constant, thermal disequilibrium conditions
*η*_td_
: Vapour adsorption per unit area of a solid under thermal disequilibrium conditions
*γ* ^LV^
: Liquid–vapour surface tension
γ
: Surface tension
*γ* ^S0^
: Solid surface tension in the absence of adsorption
*γ* ^SL^
: Solid–liquid surface tension
*γ* ^SV^
: Liquid–vapour surface tension
μ
: Chemical potential
*τ*_m_
: Measured thickness of an adsorbed film
θ
: Contact angle
ζ
: The number of molecules in a cluster
*ζ*_c_
: Critical number of molecules in a cluster
*ζ*_m_
: The maximum number of molecules in a cluster
*ζ*_th_
: The threshold number of molecules in a cluster, equilibrium condition

#### Physics constants

*k*_B_
: Boltzmann constant
*N*_A_
: Avogadro number

#### Superscripts

L: Liquid phase
LV: Liquid–vapour interface
S: Solid phase
S0: Solid surface
SV: Solid–vapour interface
V: Vapour phase

## Supplementary Material

## References

[cit1] Gao L., McCarthy T. J. (2006). Langmuir.

[cit2] Zheng Y., Gao X., Jiang L. (2007). Soft Matter.

[cit3] Sheng Q., Sun J., Wang Q., Wang W., Wang H. S. (2016). Sci. Rep..

[cit4] Kim D. E., Ahn H. S., Kwon T.-S. (2017). Appl. Therm. Eng..

[cit5] Rose J. W. (2002). Proc. Inst. Mech. Eng., Part A.

[cit6] Law K.-Y. and Zhao H., Surface Wetting: Characterization, Contact Angle, and Fundamentals, Springer, 2016

[cit7] Quéré D. (2008). Annu. Rev. Mater. Res..

[cit8] de Gennes P.-G. , Brochard-Wyart F. and Quéré D., Capillarity and Wetting Phenomena, Springer, 2004

[cit9] Bonn D., Eggers J., Indekeu J., Meunier J., Rolley E. (2009). Rev. Mod. Phys..

[cit10] Song T., Lan Z., Ma X., Bai T. (2009). Int. J. Therm. Sci..

[cit11] Khandekar S. and Muralidhar K., Dropwise Condensation on Inclined Textured Surfaces, Springer, 2014

[cit12] Tianqing L., Chunfeng M., Xiangyu S., Songbai X. (2007). AIChE J..

[cit13] Ward C. A., Wu J. (2007). J. Phys. Chem. B.

[cit14] Yaghoubian S., Ward C. A. (2017). Phys. Chem. Chem. Phys..

[cit15] Wu J., Farouk T., Ward C. A. (2007). J. Phys. Chem. B.

[cit16] Ghasemi H., Ward C. A. (2009). J. Phys. Chem. B.

[cit17] Ghasemi H., Ward C. A. (2010). J. Phys. Chem. C.

[cit18] Zandavi H., Ward C. A. (2013). J. Colloid Interface Sci..

[cit19] Zandavi S. H., Ward C. A. (2014). Phys. Chem. Chem. Phys..

[cit20] Zandavi S. H., Ward C. A. (2015). Phys. Chem. Chem. Phys..

[cit21] Zandavi S. H., Ward C. A. (2015). Energy Fuels.

[cit22] Yaghoubian S., Zandavi S. H., Ward C. A. (2016). Phys. Chem. Chem. Phys..

[cit23] Fang G., Ward C. A. (1999). Phys. Rev. E: Stat. Phys., Plasmas, Fluids, Relat. Interdiscip. Top..

[cit24] Hołyst R., Litniewski M., Jakubczyk D., Kolwas K. (2013). et al.. Rep. Prog. Phys..

[cit25] Ghasemi H., Ward C. A. (2011). J. Phys. Chem. C.

[cit26] Niu D., Tang G. H. (2016). Sci. Rep..

[cit27] Ward C. A., Wu J., Keshavarz A. (2008). J. Phys. Chem. B.

[cit28] Ward C. A., Wu J. (2008). Phys. Rev. Lett..

[cit29] Ward C. A., Tucker A. S., So C.-W. (1979). J. Phys. Chem..

[cit30] Design Institute for Physical Properties , DIPPR Project 801, Full version (electronic resource): Evaluated Standard Thermophysical Property Values, Design Institute for Physical Property Data/AIChE, 2011

[cit31] Niu D., Guo L., Hu H., Tang G. (2017). Int. J. Heat Mass Transfer.

[cit32] Lundy R., Byrne C., Bogan J., Nolan K., Collins M. N., Dalton E. (2017). ACS Appl. Mater. Interfaces.

[cit33] Preston D. J., Miljkovic N., Sack J., Enright R., Queeney J., Wang E. N. (2014). Appl. Phys. Lett..

[cit34] Rykaczewski K., Paxson A. T., Staymates M., Walker M. L., Sun X., Anand S., Srinivasan S., McKinley G. H., Chinn J., Henry J., Scott J., Varanasi K. K. (2014). Sci. Rep..

[cit35] Chibowski E., Perea-Carpio R. (2002). Adv. Colloid Interface Sci..

[cit36] Zisman W. A. , in Contact Angle, Wettability and Adhesion, ed. R. F. Good, American Chemical Society, 1964, ch. 1, vol. 43, pp. 1–51

[cit37] Tucker A. S., Ward C. A. (1975). J. Appl. Phys..

[cit38] Forest T. W., Ward C. A. (1977). J. Chem. Phys..

[cit39] Forest T. W., Ward C. A. (1978). J. Chem. Phys..

